# *Viscum album*-Mediated COX-2 Inhibition Implicates Destabilization of COX-2 mRNA

**DOI:** 10.1371/journal.pone.0114965

**Published:** 2015-02-09

**Authors:** Chaitrali Saha, Pushpa Hegde, Alain Friboulet, Jagadeesh Bayry, Srinivas V. Kaveri

**Affiliations:** 1 Institut National de la Santé et de la Recherche Médicale, Unité 1138, Paris, France; 2 Université de Technologie de Compiègne, UMR CNRS 6022, Compiègne, France; 3 Centre de Recherche des Cordeliers, Equipe-Immunopathology and therapeutic immunointervention, Paris, France; 4 Sorbonne Universités, UPMC Univ Paris 06, UMR_S 1138, Paris, France; 5 Université Paris Descartes, Sorbonne Paris Cité, UMR_S 1138, Paris, France; National Cancer Center, JAPAN

## Abstract

Extensive use of *Viscum album* (VA) preparations in the complementary therapy of cancer and in several other human pathologies has led to an increasing number of cellular and molecular approaches to explore the mechanisms of action of VA. We have recently demonstrated that, VA preparations exert a potent anti-inflammatory effect by selectively down-regulating the COX-2-mediated cytokine-induced secretion of prostaglandin E2 (PGE2), one of the important molecular signatures of inflammatory reactions. In this study, we observed a significant down-regulation of COX-2 protein expression in VA-treated A549 cells however COX-2 mRNA levels were unaltered. Therefore, we hypothesized that VA induces destabilisation of COX-2 mRNA, thereby depleting the available functional COX-2 mRNA for the protein synthesis and for the subsequent secretion of PGE2. To address this question, we analyzed the molecular degradation of COX-2 protein and its corresponding mRNA in A549 cell line. Using cyclohexamide pulse chase experiment, we demonstrate that, COX-2 protein degradation is not affected by the treatment with VA whereas experiments on transcriptional blockade with actinomycin D, revealed a marked reduction in the half life of COX-2 mRNA due to its rapid degradation in the cells treated with VA compared to that in IL-1β-stimulated cells. These results thus demonstrate that VA-mediated inhibition of PGE2 implicates destabilization of COX-2 mRNA.

## Introduction

Cyclo-oxygenase-2 (COX-2) is an early response protein, up-regulated during many pathological conditions and human malignancies. It is over expressed in most of the cells upon stimulation with diverse pro-inflammatory stimuli such as pro-inflammatory cytokines, chemokines, infectious agents, bacterial lipopolysaccharide etc. COX-2 is a critical enzyme required for the biosynthesis of prostaglandin E2, one of the important molecular mediators of inflammation [1]. Two other COX isoenzymes, COX-1 and COX-3, catalyze the same kind of reaction. COX-1 is an important cyclo-oxygenase family member, and is constitutively expressed in cells and tissues, while precise functions are not known for COX-3, which is expressed only in some specific compartments including brain and spinal cord [2, 3]. The pattern of expression of COX-1 versus COX-2 further regulates their differential functions. COX-1 is constitutively and stably expressed at low levels in many tissues. This ensures a constant production of prostaglandins, which are essentially required for the maintenance of important physiological functions, such as platelet aggregation, normal renal functions and gastric mucosal protection. In contrast, COX-2 is mostly quiescent but the expression can be induced in response to diverse pro-inflammatory and pathogenic stimuli. When stimulated, its expression is high and transient which leads to a burst of prostaglandin production in a regulated time-limited manner [4]. Thus, depending on the COX isoform, the production of the same precursor PGH2 from arachidonic acid differs with respect to the amount and timing of production. This can be differentially decoded by the cells, thereby leading to the activation of various intracellular pathways involving specific classes of prostaglandins and therefore, different responses [5].

Since COX-2 expression is up-regulated during several pathological conditions and human malignancies, strategies controlling the expression and activity of COX-2 have been developed as potent anti-tumor and anti-inflammatory treatments [6–10]. In line with the therapeutic benefit of non steroid anti-inflammatory drugs (NSAID), which are synthetically designed mainly to inhibit the enzymatic activity of COX-2, a diverse spectrum of therapeutics of natural origin such as phytotherapeutics have been characterized to evaluate their potential to inhibit the COX-2 functioning thereby down-regulating the pathological level of prostaglandins. Due to the structural homology between COX-1 and COX-2, most of the NSAID inhibit both the enzymes and thus resulting in several severe side effects due to the inhibition of physiological prostaglandins. Therefore, selective inhibitors of COX-2 are of great interest. Although, a promising class of synthetic COX-2 selective inhibitors called COXIBS have been developed, their therapeutic efficacy is compromised due to various side effects [11, 12]. Interestingly, several phytotherapeutics have been shown to exert therapeutic benefit via selective inhibition of COX-2. These natural molecules have been shown to interfere with the expression and regulatory mechanisms of COX-2 to inhibit its functioning [13, 14].

*Viscum album* (VA) preparations commonly called as mistletoe extracts, are extensively used as complementary therapeutics in cancer and also in the treatment of several inflammatory pathologies [15–19]. Despite their therapeutic application for several years, the underlying mechanisms are not yet clearly understood. Several lines of evidence have revealed that these preparations exert anti-tumor activities, which involve the cytotoxic properties, induction of apoptosis, inhibition of angiogenesis and several other immunomodulatory and anti-inflammatory mechanisms [20–30]. These properties collectively define the mechanistic basis for the therapeutic benefit of VA preparations. Recently we have shown that, VA preparations exert a potent anti-inflammatory effect by selectively down-regulating the COX-2-mediated cytokine-induced secretion of prostaglandin E2 (PGE2), one of the important molecular signatures of inflammatory reactions [31]. However, the molecular mechanisms associated with the *Viscum*-mediated COX-2 inhibition are not clear. VA preparations are shown to inhibit the COX-2 protein expression without modulating its expression at mRNA level suggesting a possible effect of VA on post-transcriptional events of COX-2 regulation. Several molecules and phytotherapeutics are known to interfere with the post-transcriptional and post-translation regulation of COX-2 in order to inhibit the COX-2 expression and subsequent reduction of PGE2 [32–34]. Therefore in the current study, we investigated the post-transcriptional and post-translational regulation of COX-2 by analyzing the stability of COX-2 protein and mRNA, which can explain in part, the molecular mechanisms of *Viscum*-mediated COX-2 inhibition.

## Materials and Methods

### *Viscum album* preparations

VA Qu Spez was a kind gift from Weleda AG (Arlesheim, Switzerland). VA Qu Spez is a therapeutic preparation of *Viscum album* that grows on oak trees and is obtained as an isotonic solution of 10mg/ml formulated in 0.9% NaCl. It is free from endotoxins and contains the standardized levels of mistletoe lectins.

### Culture of A549 cells

Human lung adenocarcinoma cell line A549 was a kind gift from Dr. Maria Castedo-Delrieu, Institute Gustave Roussy, Villejuif, France. A549 cells were grown in 75 cm^2^ culture flasks in Dulbecco’s modified Eagle’s medium (DMEM) F-12 (GIBCO, Life Technologies, Grand Island, NY, USA) supplemented with 10% fetal calf serum (FCS) and 50 U/ml penicillin and 50 μg/ml of streptomycin (GIBCO). Cells are incubated at 37^°^C with 5% CO_2_ in humidified atmosphere to obtain the cells of about 80–90% confluence and used for all experiments.

### Co- and post- treatment of VA Qu Spez and induction of COX-2

Cells grown in complete medium (DMEM with 10% FCS) were harvested by trypsinisation using 0.5% trypsin (Biological Industries, Kibbutz Beit Haemek, Israel) and were seeded in 12-well culture plates (0.5×10^6^/ml cells per well). Wells containing the adherent A549 were then replenished with the complete medium containing recombinant human IL-1β (10 ng/ml) (Immuno Tools, Friesoythe, Germany). In one set of experiment VA Qu Spez is added at the time of addition of IL-1 β (co-treatment) and in another set, we add VA Qu Spez 14 hours after adding IL-1β (post-treatment) and both the sets were incubated until 18 hours at 37^°^ C and 5% CO_2_. After 18 hours of incubation cells were harvested by trypsinization and used for the analysis of COX-1/COX-2 protein by flow cytometry.

### Analysis of the degradation profile of COX-2 protein by cyclohexamide pulse chase experiment

A549 cells with an appropriate confluency were treated with IL-1β for 18 hours in the presence or absence of VA Qu Spez. To block the protein synthesis 10 μg/ml of cyclohexamide (Sigma-Aldrich, Lyon, France) was added after 90 minutes of addition of IL-1β and then cells were harvested at different time intervals as indicated to achieve a clear pattern of COX-2 degradation. At each time point, expression of remaining COX-2 protein was analyzed by intracellular labelling, by flow cytometry and further validated by western blotting.

### Analysis of COX-2 mRNA half-life by actinomycin D pulse chase experiment

A549 cells with an appropriate confluency were treated with IL-1β for 4 hours in the presence or absence of VA Qu Spez. After 4 hours, 10 μg/ml of actinomycin D (Sigma-Aldrich) was added to the cells and cells were harvested by trypsinisation at different time intervals as indicated. Expression of remaining COX-2 mRNA was analyzed by RT-PCR.

### Statistical analysis

Densitometric analysis of the immunoblots was performed using BIO-1D analysis software. Values are expressed as arbitrary units. All the observations are expressed as Mean ±SEM and analyzed using two-way ANOVA. Graph-Pad Prism 5.0 is used for all the statistical analysis. *P* values less than 0.05 were considered to be statistically significant.

## Results

### Co-treatment of A549 cell with IL-1β and *Viscum album* inhibits the cytokine-induced COX-2 expression

**Fig 1:**
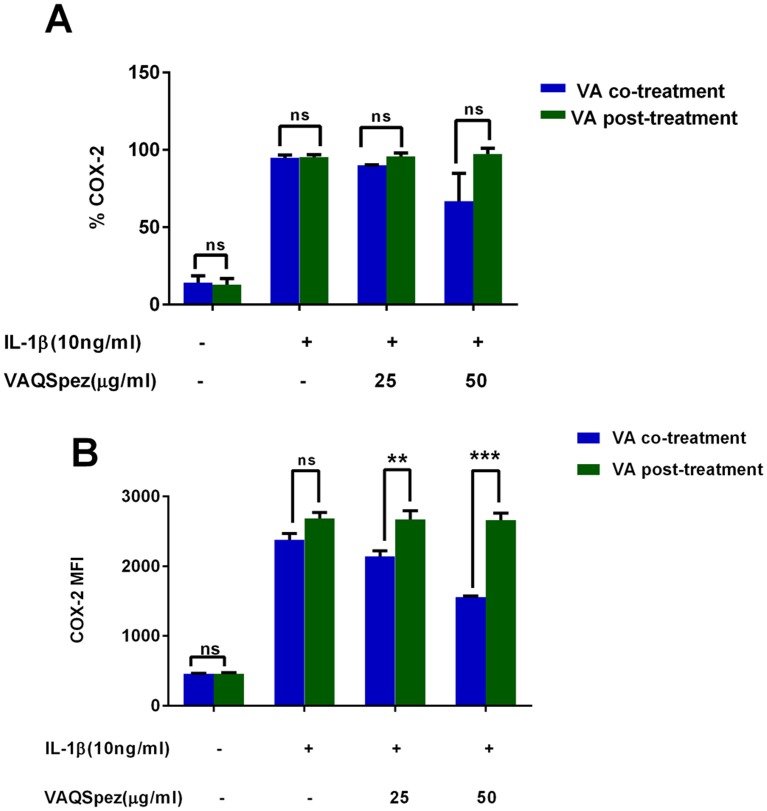
Co-treatment of A549 cell with IL-1β and *Viscum album* inhibits the cytokine-induced COX-2 expression. A549 cells were treated with IL-1β (10 ng/ml) and two different concentrations of *Viscum album* Q Spez preparation for 18 hours. Cytosolic COX-2 was measured using flow cytometric analysis. *Viscum album* is added to the cells either from the beginning of the experiment along with IL-1β (co-treatment) or after 14 hours of IL-1β induction (post-treatment). Percentage COX-2 expression as measured in intracellular staining by flow cytometry (A) and mean fluorescence intensity (MFI) of COX-2 expression (B) is shown. Results are mean ±SEM of 4 independent experiments (**p<0.01; ***p<0.001).

Following our observation of the inhibition of cytokine-induced COX-2 expression, we investigated the appropriate window of efficient inhibition by VA. Human lung adenocarcinoma (A549) cells were stimulated with IL-1β for 18 hours in the presence or absence of VA Qu Spez. VA was added to the cells either along with the cytokine (co-treatment) or after 14 hours of IL-1β induction. Flow cytometric analysis of intracellular COX-2 expression demonstrated that VA significantly inhibits cytokine-induced COX-2 expression as measured by mean fluorescent intensity (MFI) only when it is added as a co-treatment with IL-1β but not when it was added after 14 hours (Fig. 1A and 1B). This suggests that, VA-mediated COX-2 inhibition occurs at the early phases of inflammatory process and opens other exploratory avenues to understand the regulatory mechanisms of COX-2 inhibition mediated by VA at the early phase of inflammation.

### *Inhibition of COX-2 protein expression by Viscum album* is independent of modulation of stability of COX-2 protein

**Fig 2:**
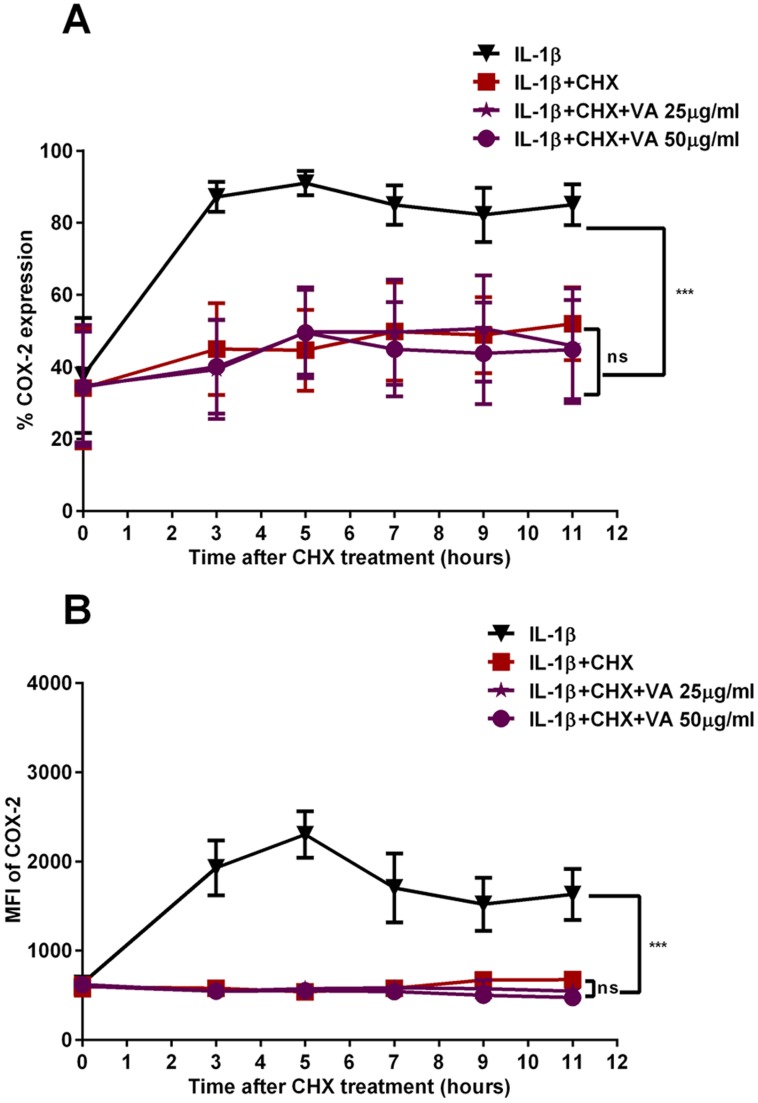
Effect of *Viscum album* on the stability of COX-2 protein as analyzed by flow cytometry. A549 cells were stimulated with IL-1β for 90 minutes with or without VA Qu Spez. Cells were harvested at different time intervals after blocking the protein synthesis with cyclohexamide (10 μg/ml) for 90 minutes till 11 hours. Normalised percentage COX-2 expression as measured in intracellular staining by flow cytometry (A) and mean fluorescence intensity (MFI) of COX-2 expression (B) is shown. Data is representative of mean ±SEM of three independent experiments.

**Fig 3:**
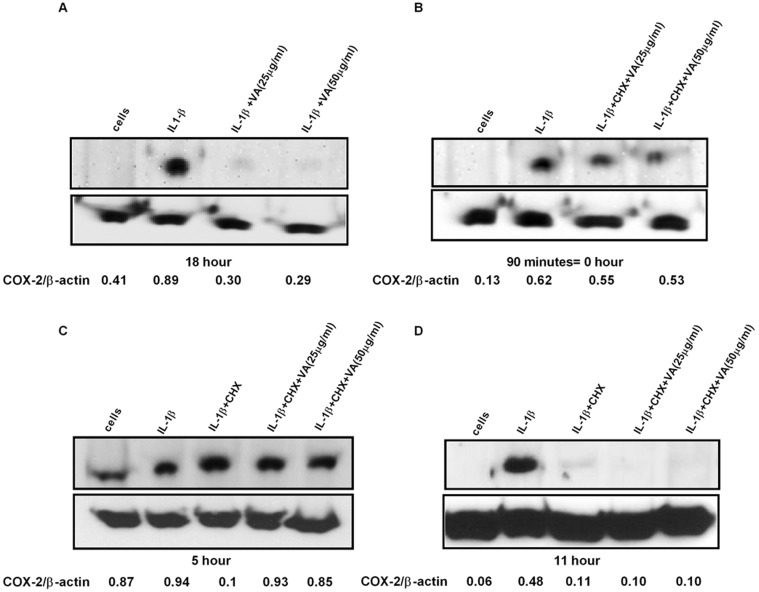
Effect of *Viscum album* on the stability of COX-2 protein as determined by western blot. Confluent A549 cells were treated with IL-1β in the presence and absence of VA Qu Spez in dose dependent concentrations in μg/ml. Cells were harvested at different time intervals after blocking the protein synthesis with cyclohexamide (10 μg/ml) for 90 minutes till 11 hours. COX-2 expression was measured by western blot using the cytosolic extracts. (A), inhibition of COX2 protein synthesis by VA at 18 hours. (B) (C) (D) are the representative western blots after 90 minutes, 5 hours and 11 hours respectively showing level of COX-2 expression after cyclohexamide treatment with or without *Viscum album*. β-actin was used as an internal control. All blots are representative of three independent experiments and the densitometry values for each band are mentioned below the representative blots.

In order to address the effect of VA on the molecular stability of COX-2, which could be a potential contributing factor for the observed reduction in COX-2 protein expression, we analyzed the stability of COX-2 protein. A549 cells were stimulated with a pro-inflammatory cytokine IL-1β in the presence and absence of VA Qu Spez. At 18 hours, we observed a significant reduction in COX-2 protein level treated with VA Qu Spez. Further, cells were harvested at different time intervals after blocking the protein synthesis by treating the cells with cyclohexamide and analyzed for COX-2. Flow cytometric analysis of COX-2 protein has revealed that, there is no significant difference in the protein degradation profile of COX-2 in VA-treated and untreated cells after 90 minutes of blocking the protein synthesis (Fig. 2A and Fig. 2B). Further, western blot analysis of COX-2 protein expression at different time intervals showed that despite the clear inhibition in the protein expression after 18 hours of exposure to cytokine followed by VA treatment (Fig. 3A), upon blocking the protein synthesis, there is no remarkable difference in the COX-2 degradation profile in cells treated with cytokine irrespective of VA treatment (Fig. 3B, 3C and 3D). Fig. 3B indicates the level of COX-2 expression immediately after 90 minutes of cyclohexamide addition (0 hour). Figs. 3C and 3D indicate the level of COX-2 expression upon blocking the protein synthesis after 5 and 11 hours respectively. These results may indicate that the regulation of COX-2 by VA may occur in an early phase of COX-2 expression but not at the later stages of protein expression and stabilization.

### *Viscum album* increases the COX-2mRNA degradation

**Fig 4:**
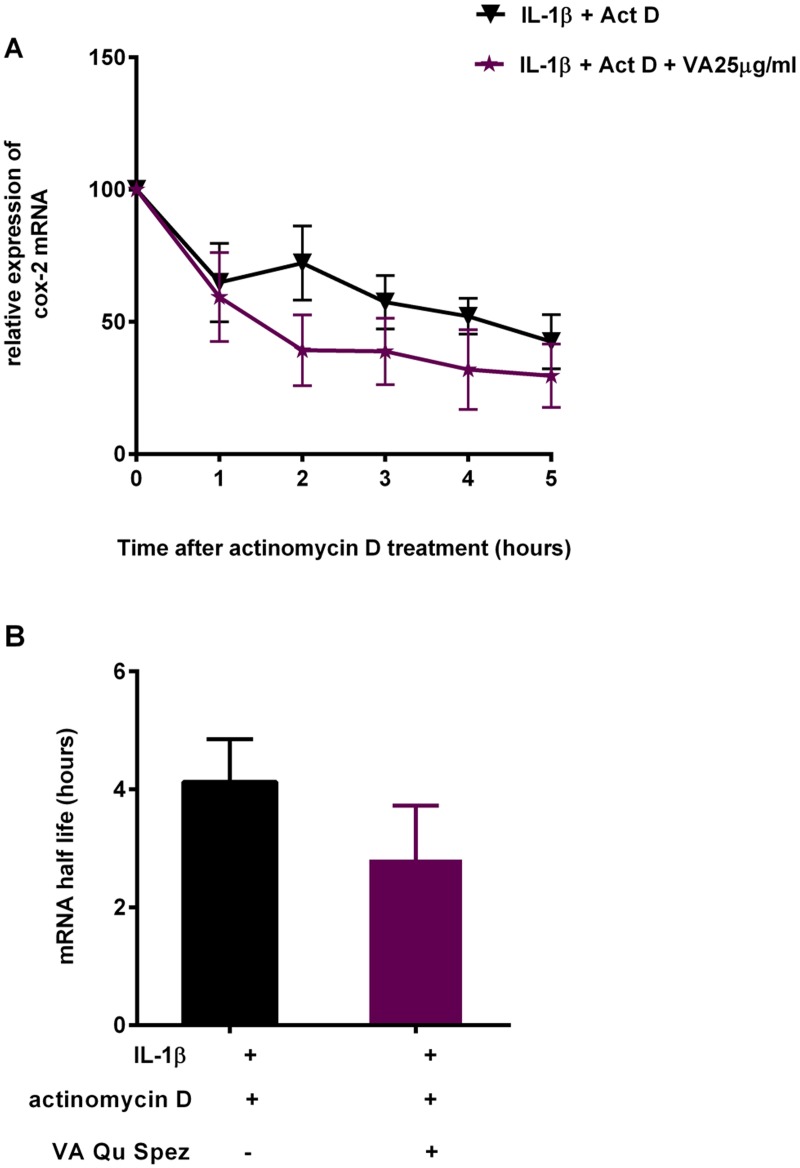
Increase in the COX-2 mRNA degradation by *Viscum album* treatment. A549 cells were stimulated with a pro-inflammatory cytokine IL-1β in the presence and absence of VA Qu Spez for 4 hours. After 4 hours of IL-1β stimulation cells are blocked with actinomycin D (10 μg/ml). Cells were harvested at different time intervals after adding actinomycin D and total cellular RNA was isolated and used for RT-PCR for the estimation of COX-2 mRNA. Relative expression of remaining COX-2 mRNA at each time point, in VA treated and untreated cells (A) and the time required for 50% of the mRNA degradation as COX-2 mRNA half life (B). Data is obtained from three independent experiments.

Due to the indication of effect of VA in the early stages of COX-2 expression, but not at the level of its mRNA expression, we analyzed the mRNA stability of COX-2 modulated by VA. A549 cells were stimulated with IL-1β in the presence and absence of VA Qu Spez for 4 hours. After 4 hours, cells were treated with actinomycin D and harvested at different time intervals. Total cellular RNA was isolated and used for RT-PCR for the estimation of COX-2 mRNA. Treatment with IL-1β is known to induce the expression of COX-2 mRNA by transcriptional activation and also by increasing the stability of COX-2 mRNA. RT-PCR analysis of COX-2 mRNA expression at different time intervals after actinomycin D treatment revealed that, at any given time interval there is a tendency to decline the relative expression of COX-2 mRNA in VA-treated cells compared to the cells treated with IL-1β (Fig. 4A). This suggests that VA at 25 μg/ml increases the rate at which the COX-2 mRNA degrades in the absence of new mRNA synthesis. Further, results from RT-PCR analysis have also showed COX-2 mRNA half life, time required for 50% of the mRNA degradation in case of VA-treated cells was marginally reduced compared to that in case of cells stimulated with cytokine alone (Fig. 4B). This suggests that VA is able to reduce the mRNA half-life of COX-2 thereby leading to its reduced bioavailability for the protein synthesis.

## Discussion

Prolonged administration of anti-inflammatory COX-2 inhibitors has been ineffective for chemopreventive and chemotherapeutic purposes since the risks prevail over the benefits. Clinical demonstration of severe side effects due to the failure of the classical COX-2 inhibitors to discriminate between an aberrant pathological versus homeostatic functional activation state, raised the concern that direct COX-2 enzymatic inhibition might not sufficiently represent an appropriate clinical strategy to target COX-2. Since in contrast to COX-1, COX-2 is an early response gene, similar to the genes encoded for cytokines, chemokines and proto-oncogenes, they can be regulated under different levels of expression and modulation, ranging from direct transcriptional effects to post-transcriptional and post-translational levels and also indirectly by various transcription factors that mediate the stability [32, 35]. Such multiple levels of modulation of COX-2 expression imply the existence of several mechanisms, which may be targeted to finely modulate COX-2 functions [36–38]. Several phytotherapeutics have been shown to exert modulatory effect on COX-2 at various levels of its molecular regulation and therefore have been considered as an effective alternative strategy to control the pathogenic expression of COX-2 [33, 39, 40]. Given that VA preparations exert a potent anti-inflammatory effect by selective down regulation of COX-2, it is extremely interesting to dissect the COX-2 inhibition mediated by VA in different regulatory mechanisms at molecular level.

Co-treatment of VA along with cytokine stimulation, marginally decreases COX-2 expression indicated by the percentage-positive COX-2 expression in Fig. 1A. However, VA significantly inhibits intensity of expression of COX-2 as analyzed by MFI. The fact that VA treatment at the later phases of cytokine induction does not inhibit COX-2 suggests that, inhibition of COX-2 by VA occurs in the early phase of COX-2 regulation but not at the later phases (Fig. 1). Since we observed an inhibition of COX-2 protein expression by VA but not of mRNA, we analyzed the protein stability of COX-2 in the presence of VA by cyclohexamide pulse chase experiments. Flow cytometric analysis of COX-2 expression after 90 minutes of blocking the protein synthesis with cyclohexamide showed that, there is no significant difference in the COX-2 degradation profile of cells simulated with IL-1β with or without treatment with VA (Fig. 2A and 2B). Western blot analysis of COX-2 protein after 5 and 11 hours of cyclohexamide blockade showed no significant difference in the degradation pattern of COX-2 in cytokine stimulated cells with or without VA treatment (Fig. 3C and 3D). Similar results at different time points were observed (data not shown). Therefore, it is clear that COX-2 protein degradation is not affected by VA. Further, reduced level of COX-2 expression at 0 hour in this experiment (Fig. 3B) also suggests that, there may be modulation by VA of the COX-2 expression before the addition of inhibitor of protein synthesis. Inhibition of COX-2 protein expression by VA (Fig. 3A) without modulating its stability (Fig. 3B, 3C and 3D) strongly indicates that, there is a possible modulation by VA at an early stage than when the proteins were expressed. However VA did not modulate COX-2 mRNA expression and therefore we analyzed the mRNA stability of COX-2 by actinomycin D pulse chase experiment. mRNA degradation profile of COX-2 obtained by analyzing the COX-2 mRNA at different time intervals after blocking the transcription using actinomycin D showed that the rate of degradation of COX-2 mRNA is higher in cells treated with VA compared to those treated with cytokine alone(Fig. 4A). This reduction in the mRNA half-life of COX-2 in the cells treated with VA (Fig. 4B) suggests that, VA induces destabilization of COX-2 mRNA, thereby diminishing the available functional mRNA for the protein synthesis and for the subsequent secretion of PGE2.

Although this study postulates destabilization of COX-2 mRNA by VA preparations as a possible mechanism for VA-mediated COX-2 inhibition, further molecular dissection is necessary in order to clearly understand the regulatory events of COX-2 regulation, contributing factors and their modulation by VA preparations.

## Conclusion

Increasing body of evidence for anti-inflammatory activity of plant-derived molecules by modulating the COX-2 functions has evolved as a potent alternative strategy for the conception of novel therapeutic molecules in the treatment of various inflammatory pathologies and in various malignancies. In view of the therapeutic benefit of VA preparations in diverse pathological situations including inflammatory and cancer conditions, dissecting their molecular mechanisms would contribute enormously to the understanding of role of phytotherapy-based treatment strategies either in complementary or alternative medicine or in other combinational therapies.

## References

[1] Rouzer CA, Marnett LJ (2009) Cyclooxygenases: structural and functional insights. J Lipid Res 50 Suppl: S29–34. 10.1194/jlr.R800042-JLR200 18952571 PMC2674713

[2] Chandrasekharan NV, Dai H, Roos KL, Evanson NK, Tomsik J, et al (2002) COX-3, a cyclooxygenase-1 variant inhibited by acetaminophen and other analgesic/antipyretic drugs: cloning, structure, and expression. Proc Natl Acad Sci U S A 99: 13926–13931. 10.1073/pnas.162468699 12242329 PMC129799

[3] Kis B, Snipes JA, Isse T, Nagy K, Busija DW (2003) Putative cyclooxygenase-3 expression in rat brain cells. J Cereb Blood Flow Metab 23: 1287–1292. 10.1097/01.WCB.0000090681.07515.81 14600435

[4] Crofford LJ (1997) COX-1 and COX-2 tissue expression: implications and predictions. J Rheumatol Suppl 49: 15–19. 9249646

[5] Kam PC, See AU (2000) Cyclo-oxygenase isoenzymes: physiological and pharmacological role. Anaesthesia 55: 442–449. 10.1046/j.1365-2044.2000.01271.x 10792135

[6] Martel-Pelletier J, Pelletier JP, Fahmi H (2003) Cyclooxygenase-2 and prostaglandins in articular tissues. Semin Arthritis Rheum 33: 155–167. 10.1016/S0049-0172(03)00134-3 14671726

[7] Zhang L, Bertucci AM, Smith KA, Xu L, Datta SK (2007) Hyperexpression of cyclooxygenase 2 in the lupus immune system and effect of cyclooxygenase 2 inhibitor diet therapy in a murine model of systemic lupus erythematosus. Arthritis Rheum 56: 4132–4141. 10.1002/art.23054 18050205

[8] Giulietti A, van Etten E, Overbergh L, Stoffels K, Bouillon R, et al (2007) Monocytes from type 2 diabetic patients have a pro-inflammatory profile. 1,25-Dihydroxyvitamin D(3) works as anti-inflammatory. Diabetes Res Clin Pract 77: 47–57. 10.1016/j.diabres.2006.10.007 17112620

[9] Zhao X, Goswami M, Pokhriyal N, Ma H, Du H, et al (2008) Cyclooxygenase-2 expression during immortalization and breast cancer progression. Cancer Res 68: 467–475. 10.1158/0008-5472.CAN-07-0782 18199541

[10] Cai Y, Lee YF, Li G, Liu S, Bao BY, et al (2008) A new prostate cancer therapeutic approach: combination of androgen ablation with COX-2 inhibitor. Int J Cancer 123: 195–201. 10.1002/ijc.23481 18386814

[11] Davenport HW (1967) Salicylate damage to the gastric mucosal barrier. N Engl J Med 276: 1307–1312. 10.1056/NEJM196706082762308 6024894

[12] Marnett LJ (2009) The COXIB experience: a look in the rearview mirror. Annu Rev Pharmacol Toxicol 49: 265–290. 10.1146/annurev.pharmtox.011008.145638 18851701

[13] Chrubasik S, Kunzel O, Model A, Conradt C, Black A (2001) Treatment of low back pain with a herbal or synthetic anti-rheumatic: a randomized controlled study. Willow bark extract for low back pain. Rheumatology (Oxford) 40: 1388–1393. 11752510 10.1093/rheumatology/40.12.1388

[14] Cravotto G, Boffa L, Genzini L, Garella D (2010) Phytotherapeutics: an evaluation of the potential of 1000 plants. J Clin Pharm Ther 35: 11–48. 10.1111/j.1365-2710.2009.01096.x 20175810

[15] Bock PR, Friedel WE, Hanisch J, Karasmann M, Schneider B (2004) [Efficacy and safety of long-term complementary treatment with standardized European mistletoe extract (Viscum album L.) in addition to the conventional adjuvant oncologic therapy in patients with primary non-metastasized mammary carcinoma. Results of a multi-center, comparative, epidemiological cohort study in Germany and Switzerland]. Arzneimittelforschung 54: 456–466. 15460213 10.1055/s-0031-1296999

[16] Klopp R, Schmidt W, Werner E, Werner M, Niemer W, et al (2005) Influence of complementary Viscum album (Iscador) administration on microcirculation and immune system of ear, nose and throat carcinoma patients treated with radiation and chemotherapy. Anticancer Res 25: 601–610. 15816634

[17] Christen-Clottu O, Klocke P, Burger D, Straub R, Gerber V (2010) Treatment of clinically diagnosed equine sarcoid with a mistletoe extract (Viscum album austriacus). J Vet Intern Med 24: 1483–1489. 10.1111/j.1939-1676.2010.0597.x 21039860

[18] Kienle GS, Kiene H (2010) Review article: Influence of Viscum album L (European mistletoe) extracts on quality of life in cancer patients: a systematic review of controlled clinical studies. Integr Cancer Ther 9: 142–157. 10.1177/1534735410369673 20483874

[19] Tusenius KJ, Spoek AM, van Hattum J (2005) Exploratory study on the effects of treatment with two mistletoe preparations on chronic hepatitis C. Arzneimittelforschung 55: 749–753. 16430029 10.1055/s-0031-1296925

[20] Bussing A, Bischof M, Hatzmann W, Bartsch F, Soto-Vera D, et al (2005) Prevention of surgery-induced suppression of granulocyte function by intravenous application of a fermented extract from Viscum album L. in breast cancer patients. Anticancer Res 25: 4753–4757. 16334172

[21] Bussing A, Schietzel M (1999) Apoptosis-inducing properties of Viscum album L. extracts from different host trees, correlate with their content of toxic mistletoe lectins. Anticancer Res 19: 23–28. 10226520

[22] Duong Van Huyen JP, Bayry J, Delignat S, Gaston AT, Michel O, et al (2002) Induction of apoptosis of endothelial cells by Viscum album: a role for anti-tumoral properties of mistletoe lectins. Mol Med 8: 600–606. 12477970 PMC2039938

[23] Duong Van Huyen JP, Delignat S, Bayry J, Kazatchkine MD, Bruneval P, et al (2006) Interleukin-12 is associated with the in vivo anti-tumor effect of mistletoe extracts in B16 mouse melanoma. Cancer Lett 243: 32–37. 10.1016/j.canlet.2005.11.016 16412563

[24] Duong Van Huyen JP, Delignat S, Kazatchkine MD, Kaveri SV (2003) Comparative study of the sensitivity of lymphoblastoid and transformed monocytic cell lines to the cytotoxic effects of Viscum album extracts of different origin. Chemotherapy 49: 298–302. 10.1159/000074530 14671430

[25] Duong Van Huyen Sooryanarayana JP, Delignat S, Bloch MF, Kazatchkine MD, et al (2001) Variable sensitivity of lymphoblastoid cells to apoptosis induced by Viscum album Qu FrF, a therapeutic preparation of mistletoe lectin. Chemotherapy 47: 366–376. 10.1159/000048545 11561140

[26] Elluru S, Duong Van Huyen JP, Delignat S, Prost F, Bayry J, et al (2006) Molecular mechanisms underlying the immunomodulatory effects of mistletoe (Viscum album L.) extracts Iscador. Arzneimittelforschung 56: 461–466. 16927527 10.1055/s-0031-1296813

[27] Elluru SR, Duong van Huyen JP, Delignat S, Kazatchkine MD, Friboulet A, et al (2008) Induction of maturation and activation of human dendritic cells: a mechanism underlying the beneficial effect of Viscum album as complimentary therapy in cancer. BMC Cancer 8: 161 10.1186/1471-2407-8-161 18533025 PMC2442603

[28] Elluru SR, Duong Van Huyen JP, Delignat S, Prost F, Heudes D, et al (2009) Antiangiogenic properties of viscum album extracts are associated with endothelial cytotoxicity. Anticancer Res 29: 2945–2950. 19661299

[29] Hostanska K, Hajto T, Spagnoli GC, Fischer J, Lentzen H, et al (1995) A plant lectin derived from Viscum album induces cytokine gene expression and protein production in cultures of human peripheral blood mononuclear cells. Nat Immun 14: 295–304. 8933823

[30] Lavastre V, Cavalli H, Ratthe C, Girard D (2004) Anti-inflammatory effect of Viscum album agglutinin-I (VAA-I): induction of apoptosis in activated neutrophils and inhibition of lipopolysaccharide-induced neutrophilic inflammation in vivo. Clin Exp Immunol 137: 272–278. 10.1111/j.1365-2249.2004.02545.x 15270843 PMC1809108

[31] Hegde P, Maddur MS, Friboulet A, Bayry J, Kaveri SV (2011) Viscum album exerts anti-inflammatory effect by selectively inhibiting cytokine-induced expression of cyclooxygenase-2. PLoS One 6: e26312 10.1371/journal.pone.0026312 22028854 PMC3196571

[32] Ristimaki A, Garfinkel S, Wessendorf J, Maciag T, Hla T (1994) Induction of cyclooxygenase-2 by interleukin-1 alpha. Evidence for post-transcriptional regulation. J Biol Chem 269: 11769–11775. 8163473

[33] Cerella C, Sobolewski C, Dicato M, Diederich M (2010) Targeting COX-2 expression by natural compounds: a promising alternative strategy to synthetic COX-2 inhibitors for cancer chemoprevention and therapy. Biochem Pharmacol 80: 1801–1815. 10.1016/j.bcp.2010.06.050 20615394

[34] Tong X, Van Dross RT, Abu-Yousif A, Morrison AR, Pelling JC (2007) Apigenin prevents UVB-induced cyclooxygenase 2 expression: coupled mRNA stabilization and translational inhibition. Mol Cell Biol 27: 283–296. 10.1128/MCB.01282-06 17074806 PMC1800648

[35] Tamura M, Sebastian S, Yang S, Gurates B, Fang Z, et al (2002) Interleukin-1beta elevates cyclooxygenase-2 protein level and enzyme activity via increasing its mRNA stability in human endometrial stromal cells: an effect mediated by extracellularly regulated kinases 1 and 2. J Clin Endocrinol Metab 87: 3263–3273. 10.1210/jcem.87.7.8594 12107235

[36] Tetsuka T, Baier LD, Morrison AR (1996) Antioxidants inhibit interleukin-1-induced cyclooxygenase and nitric-oxide synthase expression in rat mesangial cells. Evidence for post-transcriptional regulation. J Biol Chem 271: 11689–11693. 8662662 10.1074/jbc.271.20.11689

[37] Chun KS, Surh YJ (2004) Signal transduction pathways regulating cyclooxygenase-2 expression: potential molecular targets for chemoprevention. Biochem Pharmacol 68: 1089–1100. 10.1016/j.bcp.2004.05.031 15313405

[38] Surh YJ, Kundu JK (2005) Signal transduction network leading to COX-2 induction: a road map in search of cancer chemopreventives. Arch Pharm Res 28: 1–15. 10.1007/BF02975128 15742801

[39] Kundu JK, Na HK, Chun KS, Kim YK, Lee SJ, et al (2003) Inhibition of phorbol ester-induced COX-2 expression by epigallocatechin gallate in mouse skin and cultured human mammary epithelial cells. J Nutr 133: 3805S–3810S. 14608118 10.1093/jn/133.11.3805S

[40] Shrotriya S, Kundu JK, Na HK, Surh YJ (2010) Diallyl trisulfide inhibits phorbol ester-induced tumor promotion, activation of AP-1, and expression of COX-2 in mouse skin by blocking JNK and Akt signaling. Cancer Res 70: 1932–1940. 10.1158/0008-5472.CAN-09-3501 20179211

